# Optoelectronic Properties and Temporal Stability of AZO/Al/Cu/Al/AZO Multilayer Films

**DOI:** 10.3390/nano16171046

**Published:** 2026-08-22

**Authors:** Haijuan Mei, Libin Gan, Rui Wang, Jianchu Liang, Yi Yu, Yuhao Luo, Jiayu Chen, Shanshan Chen, Cihong Lin, Qiuguo Li, Weiping Gong

**Affiliations:** 1 Guangdong Provincial Key Laboratory of Electronic Functional Materials and Devices, Huizhou University, Huizhou 516007, China; 2 Guangxi Key Laboratory of Special Engineering Equipment and Control, Guilin University of Aerospace Technology, Guilin 541004, China; 3 Department of Materials Science and Engineering, Southern University of Science and Technology, Shenzhen 518000, China

**Keywords:** AZO/Cu/AZO, interfacial modulation, optoelectronic properties, temporal stability

## Abstract

To investigate how the position and thickness of ultrathin Al interfacial layers regulate the optoelectronic properties and temporal stability of AZO/Cu/AZO multilayer films, AZO/Cu/AZO (ACA), AZO/Al/Cu/AZO (AACA), and AZO/Al/Cu/Al/AZO (AACAA) multilayers were deposited on glass substrates by magnetron sputtering. For clarity, the stack notation is given from the film surface toward the substrate. AACA contains a 1 nm Al interfacial layer above Cu, whereas AACAA-1 and AACAA-2 contain Al layers on both sides of Cu with top/bottom thicknesses of 1/1 and 2/1 nm, respectively. The effects of Al layer insertion position and thickness on the microstructure, optoelectronic properties, and temporal stability were systematically investigated. The ACA and AACA films exhibited ZnO and Cu phases with preferred ZnO (002) and Cu (111) diffraction, respectively. The AACA film showed the best initial optoelectronic performance, with the average transmittance increasing from 75.9% to 85.7% and the sheet resistance decreasing from 18.5 to 6.7 Ω/sq, yielding a figure of merit (*F_OM_*) of 3.2 × 10^−2^ Ω^−1^. After the additional Al layer was introduced beneath Cu, the Cu (111) signal became very weak and the sheet resistance increased markedly, indicating a substantial change in the structural and interfacial state of the ultrathin Cu layer. After two years of air exposure, pronounced Cu-O-rich particles were observed on the ACA surface, and the relative changes in average transmittance and sheet resistance reached 10.7% and 95.7%, respectively. In contrast, the corresponding changes for AACAA-2 were only 1.2% and 2.7%, demonstrating the best temporal stability. These results reveal a clear trade-off between initial optoelectronic performance and long-term stability and show that dual Al interfacial modification is an effective route for stabilizing AZO/Cu/AZO multilayer electrodes.

## 1. Introduction

Transparent conductive oxides (TCOs) combine high visible-light transmittance with good electrical conductivity and have broad application prospects and considerable research value in optoelectronic fields such as solar cells, flat-panel displays, light-emitting diodes, and flexible electronics [1,2,3,4]. As transparent electrode materials, TCOs not only play a central role in carrier injection and transport but also enable optical coupling for light input and output, and have therefore been widely used in optoelectronic devices. Among various TCO materials, indium tin oxide (ITO) films exhibit high optical transmittance and electrical conductivity, and their structural and optoelectronic properties can be regulated by adjusting the deposition temperature and film thickness [5]. However, ITO still suffers from several limitations, including the relatively high cost of raw materials, considerable mechanical brittleness, and restricted applicability in flexible devices [6]. Compared with ITO, aluminum-doped ZnO (AZO) offers the advantages of abundant raw materials, low cost, environmental friendliness, and high optical transmittance, and is therefore considered a promising transparent conductive film [7]. Nevertheless, the resistivity of single-layer AZO films generally remains on the order of 10^−3^ Ω·cm, making it difficult to simultaneously satisfy the requirements of high transmittance and low resistance for high-performance transparent electrodes.

To overcome the performance limitations of single-layer TCO films, ultrathin metal layers have been incorporated into oxide films to construct TCO/metal/TCO (TMT) multilayer structures. TCO/Ag/TCO multilayer films have been applied to CIGS solar cells and have demonstrated promising application potential [8]. In a TMT structure, the ultrathin intermediate metal layer mainly provides an efficient carrier-transport pathway, whereas the TCO layers on both sides suppress light reflection from the metal layer and enhance optical transmittance through destructive interference, thereby achieving a balance between transmittance and electrical conductivity [9,10]. Typical structures include AZO/Au/AZO [11], AZO/Ag/AZO [12,13], and AZO/Cu/AZO [14,15,16]. Among the candidate metals, Cu possesses relatively high electrical conductivity, abundant reserves, and a comparatively low cost, making it an important candidate material for TMT transparent electrodes. However, Ag or Cu layers are susceptible to atomic migration, agglomeration, and oxidation during film deposition, heat treatment, and long-term service, which can degrade the metallic conduction pathways and consequently deteriorate the optoelectronic properties of the films [17]. To improve the interfacial state between the metallic functional layer and the TCO layers, ultrathin interfacial layers composed of Ni, Cr, Ti, or Al are commonly introduced between them, as exemplified by AZO/Ti/Ag/AZO [18], AZO/Ti/Cu/AZO [19], and WZO/Cr/Cu/Cr/WZO [20]. Zhang et al. [21] introduced a Ni interfacial layer into an AZO/Ag/AZO structure, which effectively inhibited the diffusion of Ag atoms. After thermal oxidation at 500 °C, the film retained a transmittance of approximately 75% and a sheet resistance of approximately 7 Ω/sq, demonstrating substantially improved high-temperature stability. The introduction of a Ti interfacial layer can not only regulate the crystal growth of the upper AZO layer and improve the optoelectronic and infrared properties of AZO/Ti/Cu/AZO films, but also retard the oxidation of the Cu layer and enhance the temporal stability of the films [22]. Previous studies on AZO/Al/Cu/AZO films have shown that the Al interfacial layer exerts a thickness-dependent effect on the crystallization behavior and optoelectronic properties of the films, while an appropriate increase in the Al-layer thickness can further improve temporal stability [23]. In addition, Zhou et al. [24] introduced dual Al interfacial layers into the AZO/Ag/AZO system to construct an AZO/Al/Ag/Al/AZO five-layer structure, which effectively suppressed the diffusion of Ag atoms into the AZO layers at elevated temperatures and significantly improved the high-temperature stability of the films.

Al was selected as the interfacial modifier in the present AZO/Cu/AZO system for both material and experimental-design reasons. Our previous study showed that an ultrathin Al layer located above Cu can markedly improve the optoelectronic performance and temporal stability of AZO/Cu/AZO films [23]. In addition, Al has a strong affinity for oxygen and can form Al–O-rich passivating regions, which is favorable for limiting oxygen transport and interfacial oxidation [25,26]. Using Al on both sides of the Cu layer also makes it possible to examine the effect of insertion position without introducing a second interfacial material, thereby reducing composition-related variables when comparing single- and dual-interfacial-layer structures.

Previous work from our group on AZO/Al/Cu/AZO established the thickness-dependent behavior of a single Al interfacial layer located above Cu [23], whereas the related WZO/Al/Cu/Al/WZO study focused on the effect of Cu-layer thickness in a dual-Al architecture and on the resulting microstructure and optoelectronic properties [27]. Those studies did not isolate the specific role of adding a second Al layer beneath Cu in an AZO-based stack while simultaneously comparing initial optoelectronic performance with long-term ambient stability. Therefore, in the present study, ACA, single-top-Al AACA, and dual-Al AACAA multilayer films were prepared with the same Cu and AZO thicknesses, and their as-deposited properties were compared with those measured after two years of air exposure. By varying the insertion position and the asymmetric thickness of the Al layers, this work specifically evaluates how the additional bottom Al layer changes the structural state of the Cu/AZO interfaces and clarifies the resulting performance–stability trade-off between the AACA and AACAA designs.

## 2. Experimental Details

### 2.1. Film Deposition

**Figure 1:**
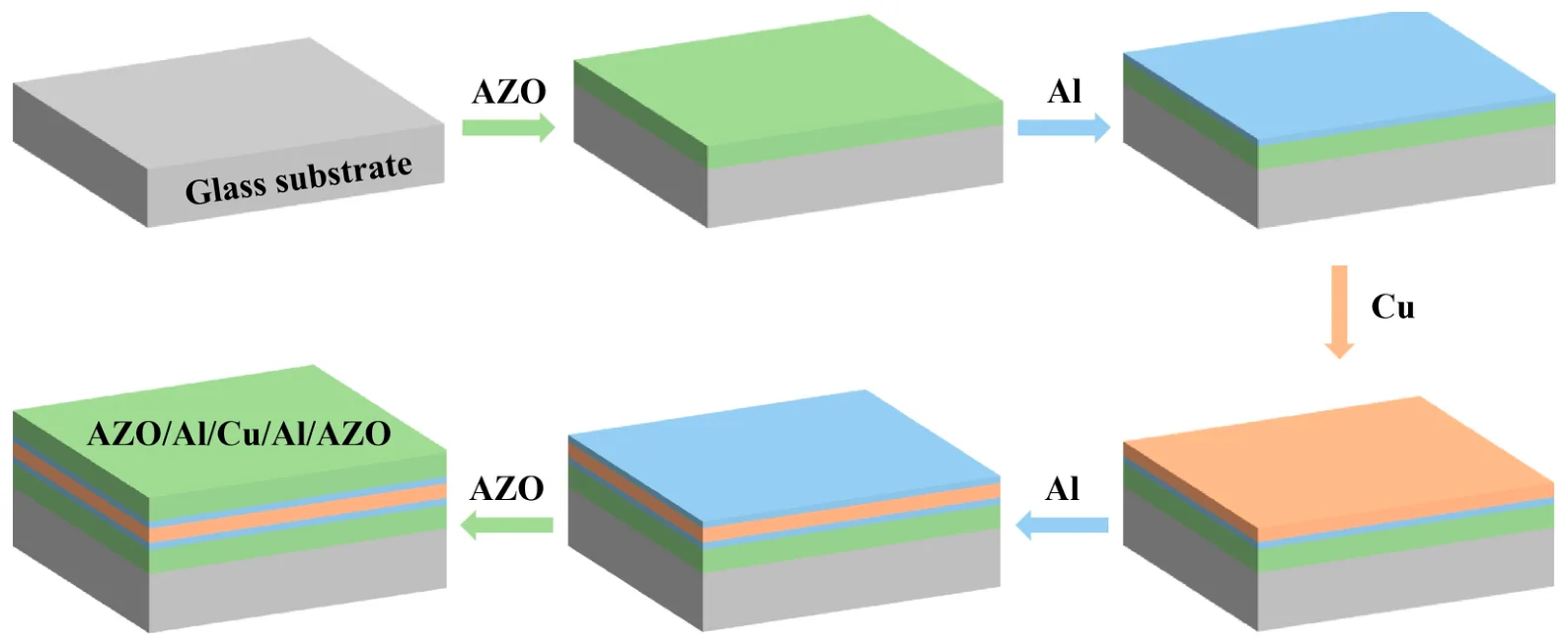
Schematic diagram of multilayer film deposition.

The AZO/Cu/AZO (ACA), AZO/Al/Cu/AZO (AACA), and AZO/Al/Cu/Al/AZO (AACAA) multilayer films were deposited on conventional glass substrates by direct-current (DC) and radio-frequency (RF) magnetron sputtering. The sputtering targets included an AZO target with a ZnO:Al_2_O_3_ weight ratio of 98:2 wt%, an Al target, and a Cu target. All targets had a purity of 99.99% and dimensions of Ø76.2 × 4 mm^2^. The multilayer films were deposited sequentially using a magnetron sputtering system, as schematically shown in Figure 1. Before deposition, all glass substrates were sequentially ultrasonically cleaned in acetone and absolute ethanol for 20 min each, dried, and then mounted on the substrate holder. The deposition chamber was evacuated to a base pressure of 8.0 × 10^−4^ Pa using mechanical and molecular pumps, after which the substrate was heated to 200 °C. High-purity Ar gas was then introduced, and the working pressure was adjusted to 0.5 Pa. Both the bottom and top AZO layers were deposited by RF magnetron sputtering at a sputtering power of 70 W for 14.5 min. The intermediate Cu layer was deposited by DC magnetron sputtering at a sputtering power of 30 W for 52 s. The interfacial Al layers were deposited by RF magnetron sputtering at a sputtering power of 300 W for 0, 5, and 10 s, corresponding to thicknesses of 0, 1, and 2 nm, respectively. The detailed deposition parameters are listed in Table 1. Multilayer films with different structures were obtained by controlling the deposition time and insertion position of the interfacial Al layers, and the thicknesses of the individual layers and the resulting multilayer films are summarized in Table 2. For clarity, the multilayer notation and the layer sequence in Table 2 are written from the film surface toward the glass substrate, whereas the actual deposition proceeds from the substrate upward as shown in Figure 1. Accordingly, AACA contains a 1 nm top Al layer above Cu, AACAA-1 contains 1 nm Al layers above and below Cu, and AACAA-2 contains a 2 nm top Al layer and a 1 nm bottom Al layer.

### 2.2. Film Characterization

Surface morphology of the films was examined using a scanning electron microscope (SEM, Vega3, Tescan, Brno, Czech Republic), and the film thickness was determined from cross-sectional images. The elemental distribution of the films was analyzed using an electron probe microanalyzer (EPMA, EPMA-1720, Shimadzu, Kyoto, Japan). The phase structure of the films was characterized by X-ray diffraction (XRD, MiniFlex 600, Rigaku, Tokyo, Japan) in the conventional θ–2θ mode. After Gaussian fitting of the diffraction peaks, the grain size, lattice constant, and residual stress were calculated using the Scherrer equation [28], Bragg’s law [16], and the biaxial strain model [29], respectively. The nanoscale multilayer structure was examined using a transmission electron microscope (TEM, Talos F200X, Thermo, Brno, Czech Republic). The TEM specimens, with a thickness of less than 100 nm, were prepared using a dual-beam focused ion beam system (FIB, Helios 5 CX, Thermo, Czech Republic). The optical transmittance of the films in the wavelength range of 300–1000 nm was measured using a transmittance–reflectance spectrophotometer (723PCSR, Ruifeng, Guangzhou, China). The sheet resistance of the films was measured using a four-point probe resistivity tester (FT-316B, Ruike, Ningbo, China), with the four probes arranged linearly at a spacing of 2.35 mm.

## 3. Results

### 3.1. Microstructure

**Figure 2:**
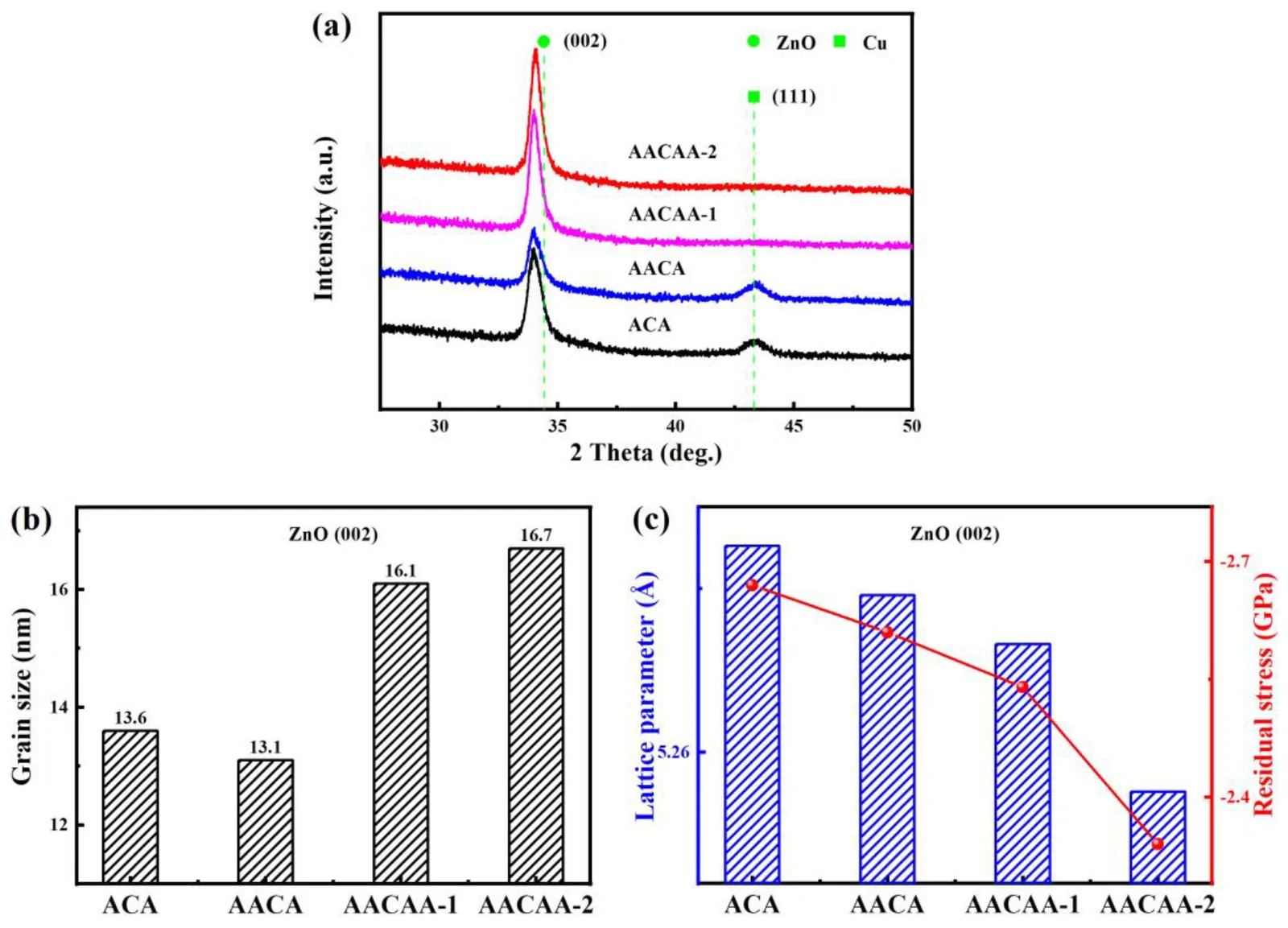
(**a**) XRD pattern of the multilayer films, (**b**) grain size, (**c**) lattice parameter and residual stress of the AZO films.

Figure 2 shows the XRD patterns, grain sizes, lattice constants, and residual stresses of the multilayer films. As shown in Figure 2a, the ACA film exhibited diffraction peaks near 34.0° and 43.3°, corresponding to the ZnO (002) and Cu (111) planes, respectively, indicating c-axis-preferred wurtzite ZnO together with face-centered-cubic Cu. No Al-related diffraction peak was observed because the nominal Al layers were only 1–2 nm thick. After a 1 nm top Al interfacial layer was introduced above Cu (AACA), the ZnO (002) intensity decreased slightly, while the Cu (111) signal remained similar to that of ACA. When an additional 1 nm bottom Al layer was introduced beneath Cu (AACAA-1), the ZnO (002) peak became much stronger, whereas the Cu (111) peak became very weak. AACAA-2 showed a similar trend. The Scherrer grain size calculated from the ZnO (002) peak first decreased slightly from 13.6 nm for ACA to 13.1 nm for AACA and then increased to 16.1 and 16.7 nm for AACAA-1 and AACAA-2, respectively. In parallel, the ZnO lattice constant and the magnitude of the residual compressive stress decreased from 5.266 Å and 2.67 GPa to 5.259 Å and 2.34 GPa, respectively. The structural evolution of the AZO layers should not be interpreted solely in terms of the relaxation of residual compressive stress. Another possible contribution is associated with the influence of the Al interfacial layer located beneath Cu on the initial growth of the ultrathin Cu layer. The bottom Al layer may modify the nucleation density, island coalescence, continuity, roughness, and local texture of the subsequently deposited Cu layer. Because the upper AZO layer nucleates directly on the Cu-related interfacial region, such changes in Cu morphology and surface structure may in turn alter the nucleation and preferred growth of the upper AZO layer. Therefore, the enhanced ZnO (002) diffraction intensity and increased grain size observed in the AACAA films may result from the combined effects of interfacial-energy modification, Cu growth morphology, local structural disorder, and stress evolution, rather than from residual-stress relaxation alone. In AACAA, however, the same top Al layer is combined with a bottom Al underlayer deposited before Cu. Such an underlayer can alter the nucleation density, wetting, coalescence, roughness, continuity, and crystallographic texture of an ultrathin Cu film. Related work has shown that Al-containing nucleation/wetting layers can reduce the percolation threshold and promote more continuous Cu growth [30], that Al-containing interlayers can be added into Cu-based dielectric/metal/dielectric electrodes [31], and that an ultrathin wetting layer can promote highly oriented two-dimensional Cu growth on ZnO [32]. Because the upper AZO is deposited on the Cu/top-Al stack, a bottom-Al-induced change in the underlying Cu morphology can therefore change the effective growth template experienced by the upper AZO, providing a plausible route to the stronger ZnO (002) response in AACAA even though the same top Al layer is present. With the present conventional θ–2θ geometry and the low diffracting volume of an 8 nm Cu layer, the signal-to-noise ratio is insufficient to distinguish quantitatively among changes in coherent-domain size, texture distribution, interfacial disorder, and film continuity. Thus, the present XRD data support only the conclusion that the dual-Al design substantially changes the structural state of the Cu-containing region.

**Figure 3:**
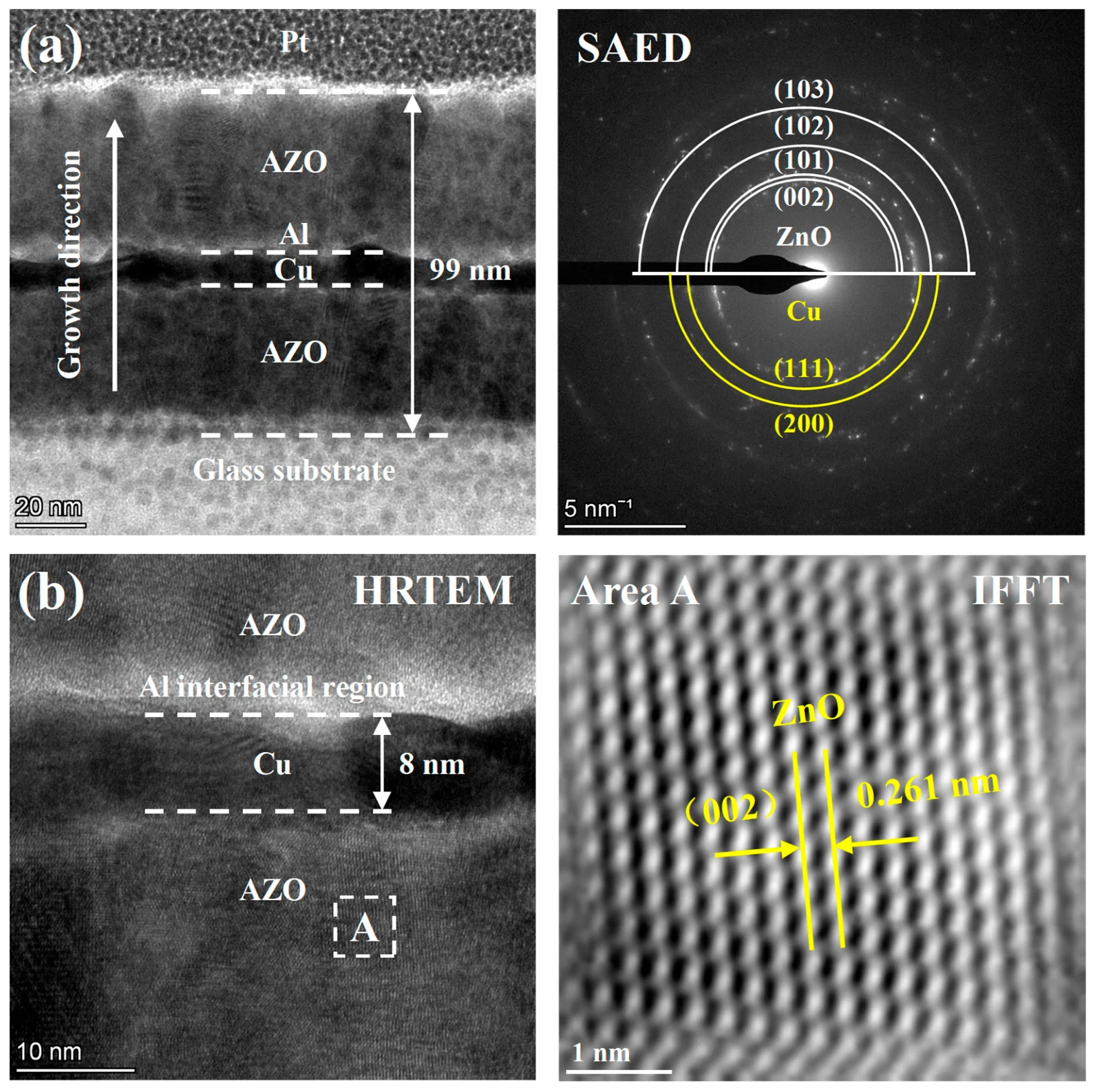
Cross-sectional TEM images of the AACA film: (**a**) bright-field image and SAED pattern, (**b**) HRTEM image and the corresponding IFFT pattern.

Figure 3 presents the TEM and HRTEM images of the AACA film. In Figure 3a, the film exhibits a nanoscale multilayer structure with distinguishable interfaces and a total thickness of approximately 99 nm. The SAED pattern contains polycrystalline diffraction rings assignable to ZnO and Cu, consistent with the phase identification from XRD. In the cross-sectional HRTEM image in Figure 3b, the dark band corresponds to the approximately 8 nm Cu layer because of the atomic-number contrast, and appears continuous within the limited cross-sectional field of view. This local observation, however, cannot establish that the buried Cu layer is completely pinhole-free over the film plane. Previous studies have demonstrated that the continuous growth state of ultrathin metal layers, together with the roughness, defects, and degree of intermixing at the metal/oxide interfaces, significantly affects the optoelectronic properties of TMT films [12]. In addition, a narrow contrast-transition region is visible between Cu and the upper AZO layer at the nominal position of the 1 nm Al deposition. This bright region corresponded to the ultrathin Al interfacial layer, which could regulate the adsorption, surface diffusion, and nucleation behavior of the deposited species at the interface, thereby affecting the crystal growth of the top AZO layer and the transfer of interfacial stress. The IFFT image obtained from area A gives a local lattice spacing of approximately 0.261 nm, which is consistent with the ZnO (002) interplanar spacing.

**Figure 4:**
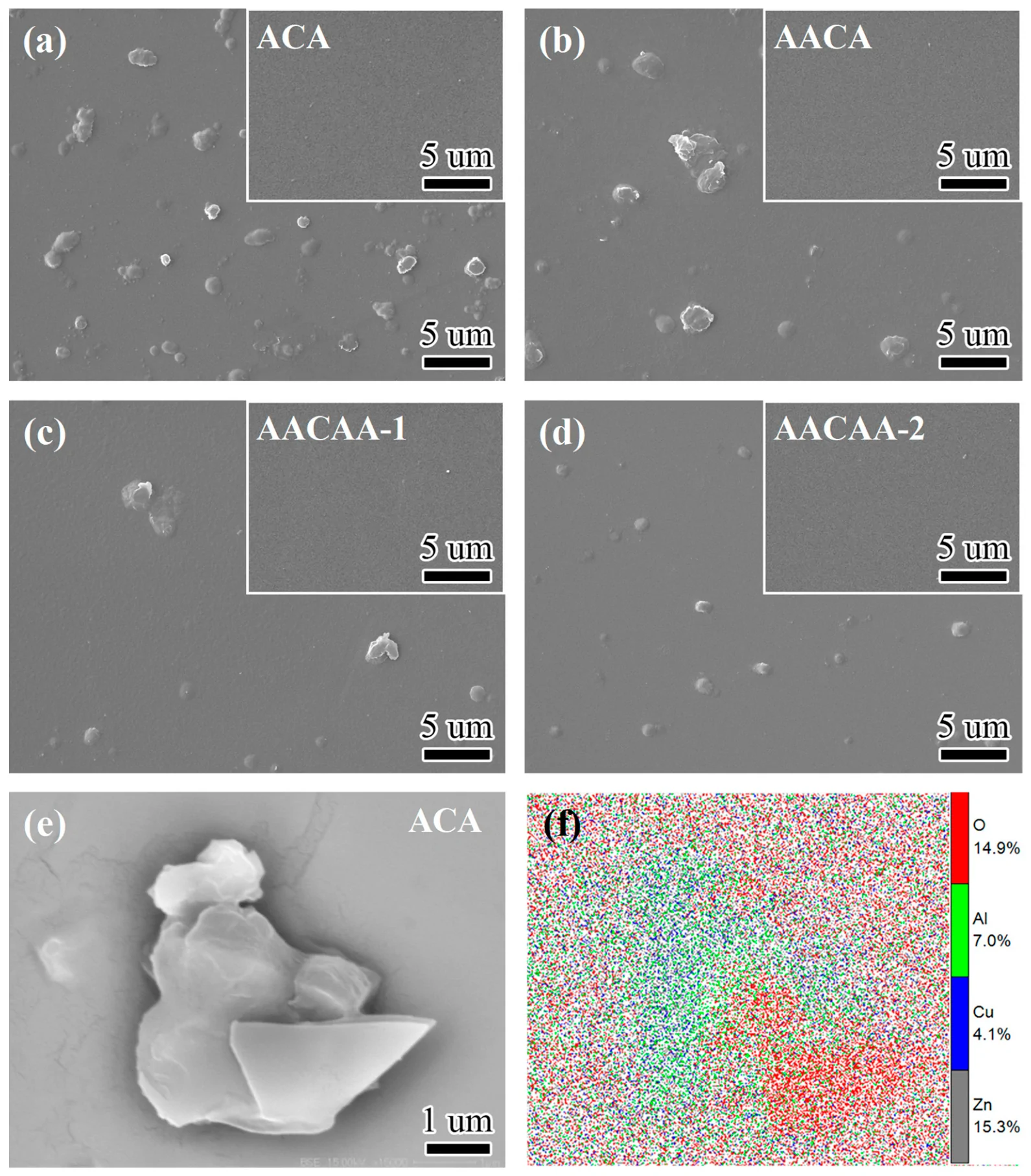
Surface SEM images of the films before and after exposure to air for 2 years: (**a**) ACA, (**b**) AACA, (**c**) AACAA-1, (**d**) AACAA-2, (**e**) oxidation particle on the surface of the ACA film, and (**f**) surface scanning.

Figure 4 compares the surface SEM morphologies of the as-deposited films and the films after two years of air exposure. As shown in the inset images, all as-deposited samples exhibited relatively smooth surfaces. After two years, pronounced large particles appeared on the ACA surface, and EPMA mapping showed that these particles were enriched in Cu and O, as shown in Figure 4e,f. The observation is consistent with outward migration of Cu through grain boundaries, defects, and interfaces in the AZO overlayer, followed by local accumulation and oxidation at the air-facing surface. Such migration and oxidation can simultaneously increase surface scattering and disrupt the metallic conduction pathway. The AACA showed substantially fewer surface particles. In this structure, the top Al interfacial layer is located directly between Cu and the upper AZO layer and can therefore modify the dominant air-facing Cu/AZO transport pathway. Its effect is plausibly associated with a combination of physical diffusion resistance and chemical passivation because Al has a strong affinity for oxygen and can form Al–O-rich interfacial regions [25,26]. The still lower particle density in AACAA, however, cannot be assigned solely to a simple two-sided ‘sealing’ effect. Oxygen transport from the glass-substrate side is expected to be much more limited than transport from the air-facing side, whereas the bottom Al layer is deposited before Cu and can modify the way the Cu layer nucleates and coalesces. Nucleation or wetting layers have been reported to lower the percolation threshold and promote smoother or more continuous ultrathin Cu growth [30,32]. If the bottom Al underlayer produces a Cu morphology with fewer pinholes, voids, discontinuous regions, or highly curved unstable features, oxygen penetration and subsequent Cu redistribution/oxidation could be reduced through a morphology-mediated pathway. The stability of ultrathin metal films is also closely linked to their morphological integrity under environmental or thermal loading [30,33]. Accordingly, the improved long-term stability of the dual-Al structures is interpreted more cautiously as the combined result of top-side diffusion/passivation and a possible bottom-Al-mediated stabilization of the buried Cu morphology. Compared with AACAA-1, AACAA-2 contains a thicker top Al layer (2 nm rather than 1 nm) while retaining the same 1 nm bottom Al layer. The further reduction in surface-particle density may therefore be associated with strengthened top-side diffusion/passivation, together with the larger AZO crystallite size and the associated reduction in grain-boundary pathways.

### 3.2. Optoelectronic Properties and Temporal Stability

**Figure 5:**
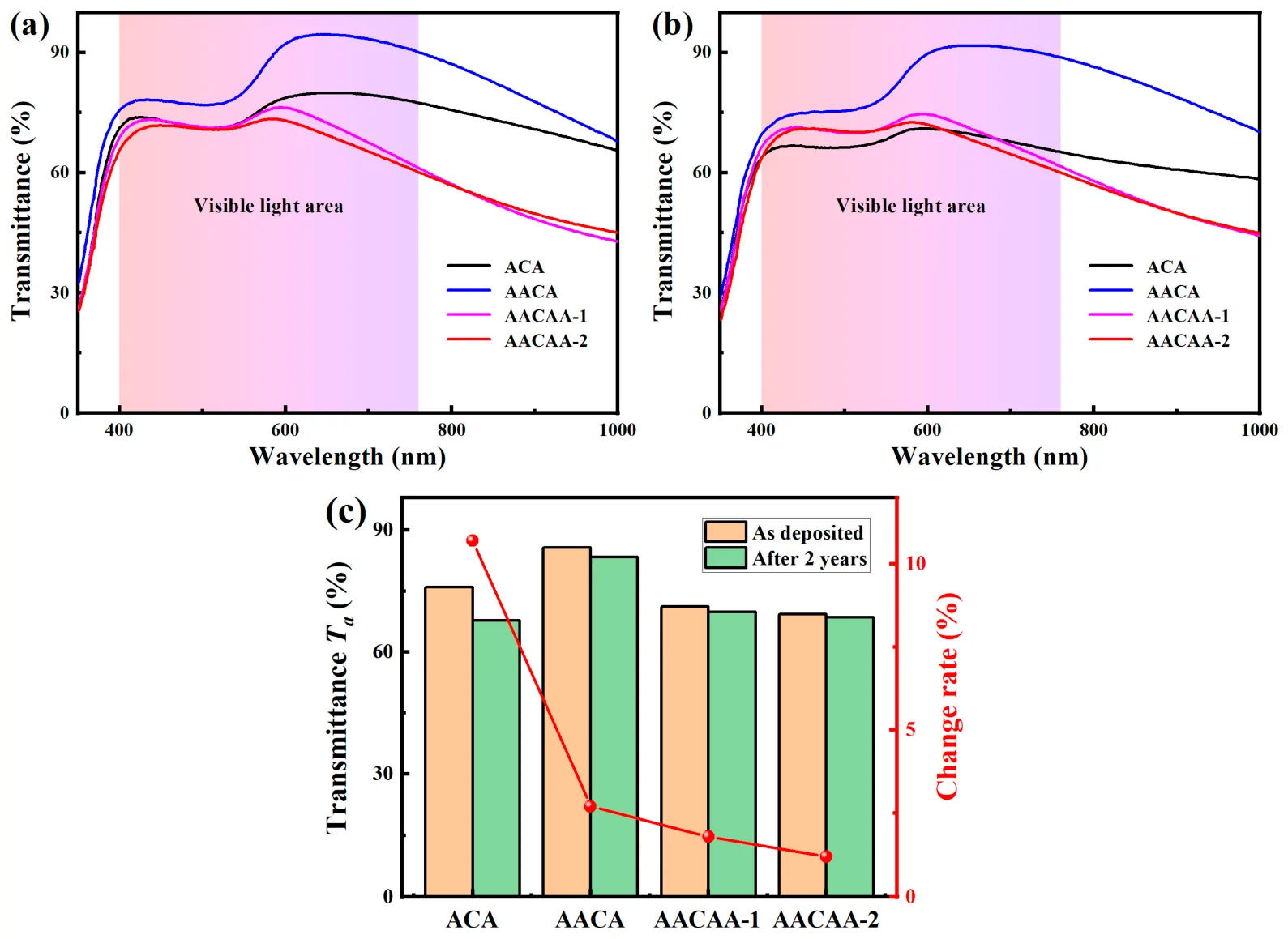
Transmittance spectra of the films before and after exposure to air for 2 years: (**a**) as deposited, (**b**) after 2 years, (**c**) average transmittance and corresponding change rate.

Figure 5 shows the transmittance spectra and average transmittance of the as-deposited films and the films after two years of air exposure. All as-deposited samples show two characteristic transmission maxima in the visible region, consistent with multiple reflection and interference in the multilayer stack. Relative to ACA, the entire AACA spectrum shifts upward and the average visible transmittance increases from 75.9% to 85.7%, even though an additional approximately 1 nm metallic Al layer is present. This substantial increase should not be interpreted as a simple consequence of adding Al, because the Al itself can introduce absorption. Rather, the most reasonable interpretation is a net optical-interface effect: the ultrathin Al interfacial region changes the effective optical admittance, optical thickness, and phase conditions at the Cu/upper-AZO side of the stack, which can reduce the net reflected intensity over part of the visible range through destructive interference [9,10,23]. In addition, nanoscale changes in interfacial roughness, mixing, or Cu morphology could modify both reflection and absorption. The Cu (111) diffraction signal remains similar between ACA and AACA, suggesting no large change in the coherent Cu diffraction response, but conventional XRD cannot exclude nanoscale changes in continuity or roughness. After the additional bottom Al layer is introduced, the AACAA-1 and AACAA-2 spectra shift downward and their average transmittances decrease to 71.2% and 69.3%, respectively. In these dual-Al structures, the added metallic absorption, the altered optical phase condition, and changes in the Cu/interfacial morphology may act simultaneously. After two years of air exposure, all spectra shift downward to different extents. The ACA film shows the largest decrease, with its average transmittance falling to 67.8% and a relative change of 10.7%. The Cu-O-rich particles observed in Figure 4 can increase both scattering and absorption. In contrast, the Al-modified structures show much smaller transmittance changes, consistent with the combined interfacial stabilization mechanisms discussed above; the minimum relative change is 1.2% for AACAA-2.

**Figure 6:**
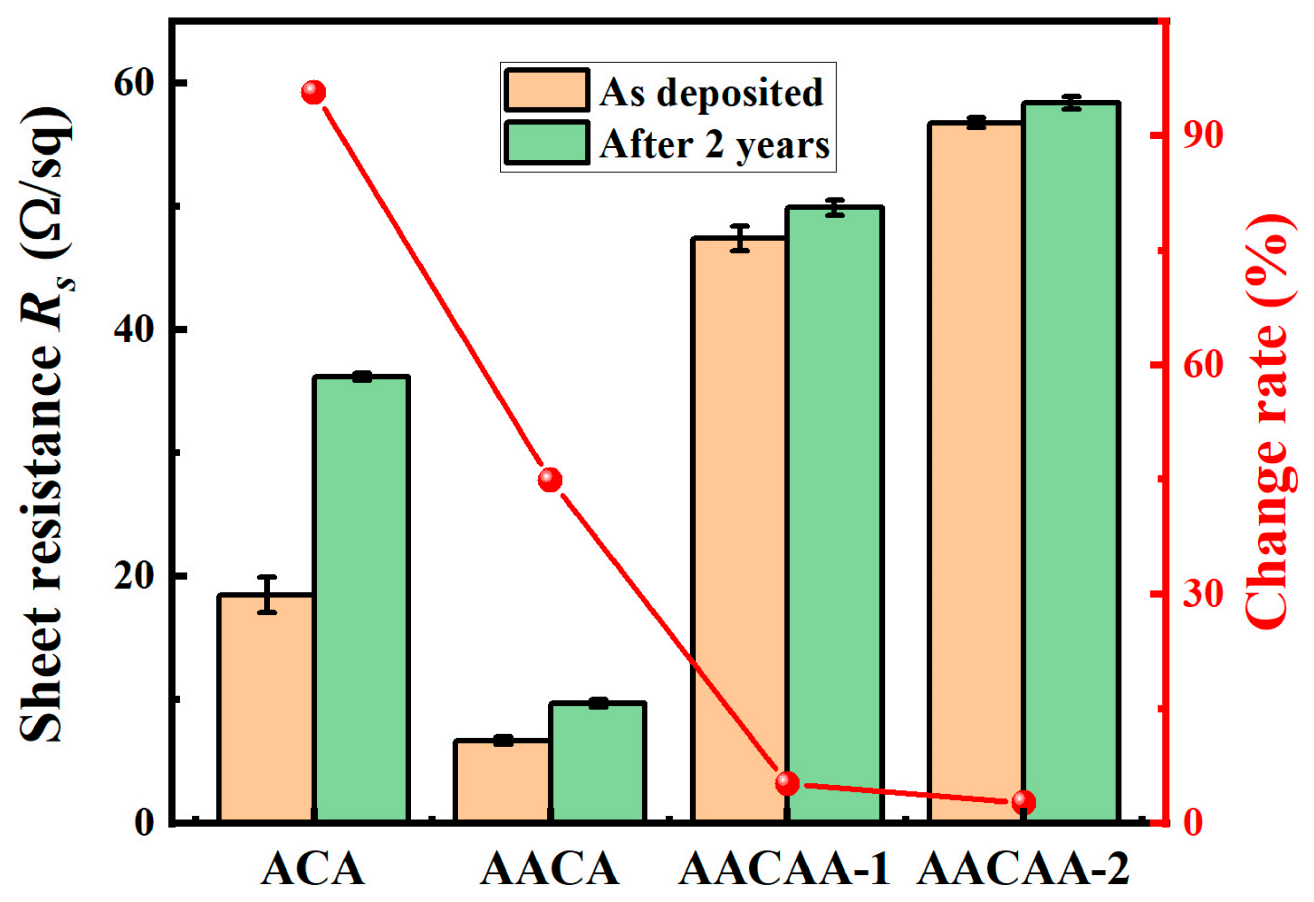
Sheet resistance of the films before and after exposure to air for 2 years and corresponding change rate.

Figure 6 presents the sheet resistance and corresponding relative changes in the as-deposited films and the films after two years of air exposure. The ACA film exhibits an initial sheet resistance of 18.5 Ω/sq, comparable to the values reported for the optimized AZO/Cu/AZO films [16]. After insertion of the 1 nm top Al layer, the AACA sheet resistance decreases to 6.7 Ω/sq. A previous study of AZO/Al/Cu/AZO showed that an appropriately thin top Al layer can increase the effective carrier concentration of the multilayer [23], and changes in the Cu/Al/AZO interfacial state and carrier-scattering pathways may also contribute. When the bottom Al layer is additionally introduced, the sheet resistance increases to 47.4 Ω/sq for AACAA-1 and 56.8 Ω/sq for AACAA-2. This increase coincides with the pronounced weakening of the Cu (111) diffraction signal, demonstrating that the dual-Al design substantially changes the Cu-containing region. The higher resistance is therefore described more cautiously as being consistent with a less favorable metallic conduction pathway and/or increased interface and defect scattering associated with the modified Cu/interfacial structure. However, after two years of air exposure, the ACA sheet resistance increases from 18.5 to 36.2 Ω/sq, corresponding to a 95.7% relative change. The Cu-O-rich surface particles and the associated outward redistribution and oxidation of Cu can reduce the effective metallic conduction cross-section and increase carrier scattering. For AACA, the relative change decreases to 44.8%, while AACAA-1 and AACAA-2 show much smaller changes of 5.2% and 2.7%, respectively. The superior retention of the dual-Al is consistent with a combination of top-side Al barrier/passivation and a possible bottom-Al-induced stabilization of the buried Cu morphology, rather than being attributed exclusively to bidirectional ‘sealing’. This interpretation is compatible with reports linking the continuity and morphological integrity of ultrathin metal films to improved environmental or thermal stability [30,33].

**Figure 7:**
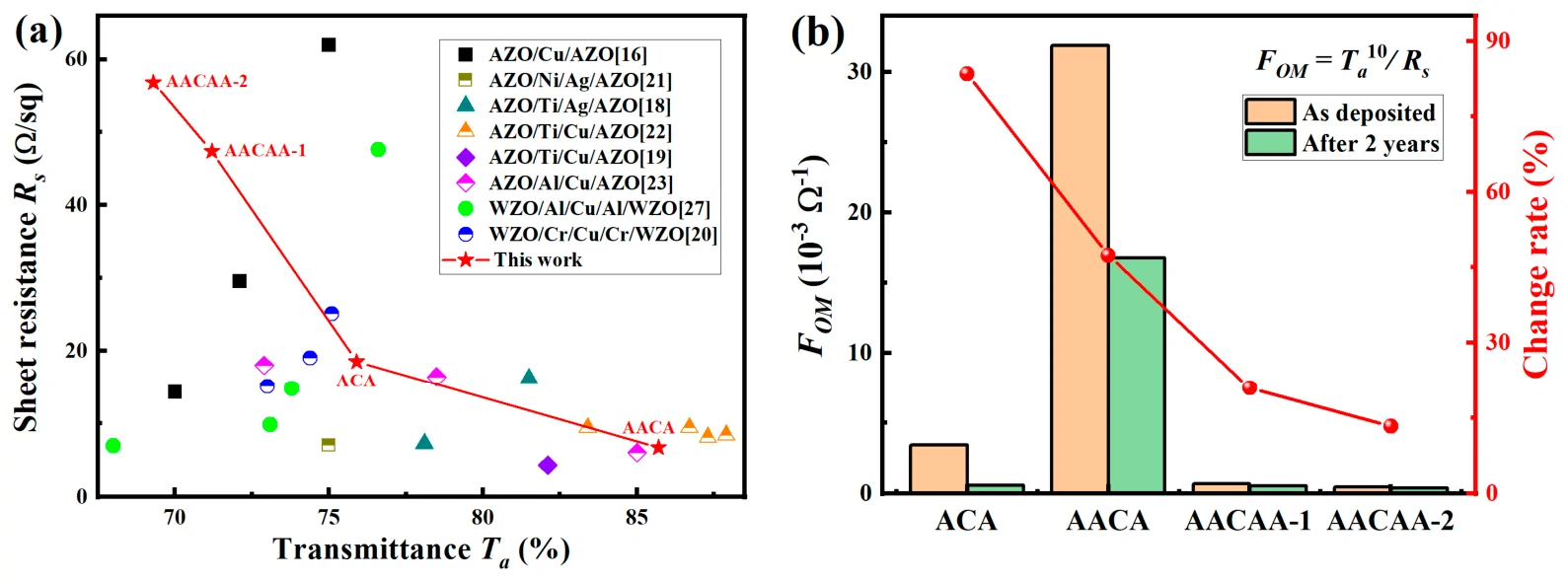
(**a**) Comparison of transmittance (*T_a_*) and sheet resistance (*R_s_*) with other studies [16,18,19,20,21,22,23,27], (**b**) figure of merit (*F_OM_*) and change rate of the films before and after exposure to air for 2 years.

Figure 7a compares the optoelectronic properties of the multilayer films prepared in this study with values reported in the literature. Ultrathin Ni, Ti, Al, and Cr interfacial layers have been used to regulate metal/oxide interfaces and improve the balance between optical transmittance and electrical conductivity. Compared with the previously reported AZO/Cu/AZO film [16], the ACA film exhibits favorable optoelectronic properties, while the AACA film shows the best initial performance after introduction of the 1 nm top Al layer. The figure of merit (*F_OM_*) is commonly used to evaluate the overall optoelectronic performance of transparent conductive films [34]. In Figure 7b, the as-deposited ACA film has an *F_OM_* of 3.4 × 10^−3^ Ω^−1^, whereas the AACA film reaches 3.2 × 10^−2^ Ω^−1^ because of its simultaneously higher transmittance and lower sheet resistance. After the bottom Al layer is additionally introduced, the *F_OM_* decreases to 7.1 × 10^−4^ and 4.5 × 10^−4^ Ω^−1^ for AACAA-1 and AACAA-2, respectively, reflecting their lower transmittance and higher sheet resistance. After two years of air exposure, the *F_OM_* values of all samples decrease, but to markedly different extents. The *F_OM_* of ACA decreases from 3.4 × 10^−3^ to 5.7 × 10^−4^ Ω^−1^, corresponding to an 83.5% relative change, whereas AACAA-2 shows the smallest relative change of only 13.3%. The progressively improved *F_OM_* retention with Al interfacial modification is consistent with reduced Cu redistribution and oxidation. For the dual-Al structures, this stabilization can be attributed to the combined top-side diffusion/passivation effect and a possible bottom-Al-mediated influence on the morphology of the buried Cu layer. These results reveal an optoelectronic property–stability trade-off. AACA provides the highest initial optoelectronic performance because its high transmittance and low sheet resistance yield the largest *F_OM_,* whereas the AACAA structures sacrifice initial transmittance and conductivity but exhibit far smaller property drift during two years of ambient exposure. From a practical design perspective, the AACA architecture is therefore more attractive when maximum transparency and conductivity are the primary requirements. By contrast, the AACAA architecture is advantageous when long-term retention of electrical and optical properties is prioritized, although its initial sheet resistance remains too high for many demanding transparent-electrode applications.

## 4. Conclusions

In this study, AZO/Cu/AZO, AZO/Al/Cu/AZO, and AZO/Al/Cu/Al/AZO multilayer films were deposited on glass substrates by RF and DC magnetron sputtering. The effects of the insertion position and thickness of the Al interfacial layers on the microstructure, optoelectronic properties, and temporal stability were systematically investigated. The results showed that both the ACA and AACA films consisted of a hexagonal wurtzite ZnO phase and a face-centered cubic Cu phase. The AACA film exhibited the best initial optoelectronic performance, with an average transmittance of 85.7%, a sheet resistance of 6.7 Ω/sq, and an *F_OM_* of 3.2 × 10^−2^ Ω^−1^. After an additional Al layer was introduced beneath Cu, the Cu (111) diffraction signal became very weak and the sheet resistance increased to 47.4 and 56.8 Ω/sq for AACAA-1 and AACAA-2, respectively. After two years of air exposure, the ACA film developed pronounced Cu-O-rich surface particles and its *F_OM_* changed by 83.5%, whereas the *F_OM_* change of AACAA-2 was only 13.3%. The top-only Al design maximizes initial optoelectronic performance, while the dual-Al design markedly improves long-term property retention at the cost of higher initial sheet resistance and lower transmittance. The enhanced stability of the dual-Al films is most reasonably attributed to a combination of top-side Al diffusion/passivation and possible bottom-Al-induced changes in Cu nucleation and morphology.

## Figures and Tables

**Table 1:** Deposition parameters of multilayer films.

Parameters			
Base pressure (Pa)	8.0 × 10^−4^		
Working temperature (°C)	200		
Working pressure (Pa)	0.5		
Ar flow rate (sccm)	150		
Target material	AZO	Al	Cu
Magnetron sputtering	RF	RF	DC
Target power (W)	70	300	30
Deposition rate (nm/min)	3.1	11.8	9.2
Deposition time	14.5 min	0, 5, 10 s	52 s

**Table 2:** Monolayer thickness and total thickness of multilayer films. The layer sequence is listed from the film surface to the glass substrate.

Film	Monolayer Thickness (nm)					Total Thickness (nm)
	AZO	Al	Cu	Al	AZO	
ACA	45	0	8	0	45	98
AACA	45	1	8	0	45	99
AACAA-1	45	1	8	1	45	100
AACAA-2	45	2	8	1	45	101

## Data Availability

The original contributions presented in this study are included in the article. Further inquiries can be directed to the corresponding authors.
